# A note on conservation laws with discontinuous flux and $L^1$ initial data

**DOI:** 10.1007/s00605-025-02105-x

**Published:** 2025-08-24

**Authors:** Kenneth H. Karlsen, Darko Mitrovic

**Affiliations:** 1 Department of mathematics, University of Oslo, P.O. Box 1053, Blindern, N–0316 Oslo Norway; 2 Faculty of Mathematics, University of Vienna, Oskar Morgenstern–Platz 1, 1090 Wien, Austria

**Keywords:** Conservation law, Discontinuous flux, $L^1$ data, Entropy condition, Kinetic formulation, Existence, Uniqueness, Primary 35L65, Secondary 35L45

## Abstract

We study conservation laws with a discontinuous flux function ${\mathfrak {f}}(\textbf{x},\lambda )$. The flux function can be expressed as $g(\beta (\textbf{x},\lambda ))$, where *g* is locally Lipschitz, $\beta (\textbf{x},\lambda )$ is an increasing function in $\lambda$ for each fixed $\textbf{x}$, $\nabla \beta (\cdot ,0)$ is a finite measure, and $\beta (\cdot ,0)$ is bounded. We consider this problem under the Audusse-Perthame entropy condition and derive a kinetic formulation. Using the kinetic approach, we prove an existence result under the assumption that the initial function belongs to $L^1$. Uniqueness results are also presented.

## Introduction

In this work, we consider the following Cauchy problem:

1.1 $$\begin{aligned} \begin{aligned}&\partial _t u +{{\,\textrm{div}\,}}_{\textbf{x}} {\mathfrak {f}}(\textbf{x},u) =0,\\&u|_{t=0} =u_0(\textbf{x}), \end{aligned} \end{aligned}$$

where the flux function ${\mathfrak {f}}$ and initial data $u_0$ satisfy the following conditions:

(i) There exist a locally Lipschitz continuous function $g:{{\mathbb {R}}}\rightarrow {{\mathbb {R}}}^d$ and a function $\beta :{{\mathbb {R}}}^d\times {{\mathbb {R}}}\rightarrow {{\mathbb {R}}}$ such that

1.2 $$\begin{aligned} \nabla \beta (\cdot ,0) \text { is a vector Radon measure on } {{\mathbb {R}}}^d, \quad \beta (\cdot ,0)\in L^\infty ({{\mathbb {R}}}^d), \end{aligned}$$

and for every $\textbf{x}\in {{\mathbb {R}}}^d$, the map $\lambda \mapsto \beta (\textbf{x},\lambda )$ is strictly increasing and onto. The vector function ${\mathfrak {f}}(\textbf{x},u)$ is defined by the relation

$$\begin{aligned} {\mathfrak {f}}(\textbf{x},u)=g(\beta (\textbf{x},u)). \end{aligned}$$

(ii) Define $\alpha (\textbf{x},\lambda ):=\beta ^{-1}(\textbf{x},\lambda )$ and set $\alpha '(\textbf{x},\lambda ):=\partial _\lambda \alpha (\textbf{x},\lambda )$. For every fixed $\lambda \in {{\mathbb {R}}}^n$, the function $\textbf{x}\mapsto \alpha (\textbf{x}, \lambda )$ belongs to $L_{\operatorname {loc}}^\infty ({{\mathbb {R}}}^d)$. Moreover, for a.e. $\textbf{x}\in {{\mathbb {R}}}^d$, the function $\lambda \mapsto \alpha (\textbf{x}, \lambda )$ belongs to $C^1([-L,L])$, and its derivative $\lambda \mapsto \alpha '(\textbf{x},\lambda )$ belongs to $C([-L,L])$, for every $L > 0$. Finally, for $|\textbf{z}|\le 1$, and $\lambda \in {{\mathbb {R}}}$,

1.3 $$\begin{aligned} \Vert \alpha '(\cdot +\textbf{z},\lambda ) -\alpha '(\cdot ,\lambda )\Vert _{L^1({{\mathbb {R}}}^d)} \le \varrho (|\textbf{z}|)p(|\lambda |), \end{aligned}$$

for some continuous increasing function $\varrho :[0,\infty )\rightarrow [0,\infty )$ with $\varrho (0)=0$, and some locally bounded function $p:[0,\infty )\rightarrow [0,\infty )$.

(iii) The initial function $u_0$ belongs to $L^1({{\mathbb {R}}}^d)$.

It follows from (iii) and the strictly increasing nature of $\lambda \mapsto \alpha (\textbf{x},\lambda )$ that

1.4 $$\begin{aligned} I_0^{\pm }(\lambda ):=\int _{{{\mathbb {R}}}^d}|\alpha (\textbf{x},\tilde{u}_0) -\alpha (\textbf{x},\lambda )|_{\pm }\,d\textbf{x}\rightarrow 0 \ \ \hbox { as}\ \lambda \rightarrow \pm \infty , \end{aligned}$$

where $\tilde{u}_0=\beta (\textbf{x},u_0)$, $|a|_{\pm }=\textrm{sgn}_{\pm }(a)a$, $\textrm{sgn}_{+}(a)=\textbf{1}_{a> 0}$, and $\textrm{sgn}_{-}(a)=-\textbf{1}_{a\le 0}$, so that we can write $a=a_+-a_-$ and $|a|=a_++a_-$. Consider the case of $I_0^+$. Given that $\lambda \mapsto \alpha (\textbf{x},\lambda )$ is strictly increasing, we can infer that

1.5 $$\begin{aligned} \lim _{\lambda \rightarrow +\infty } |\alpha (\textbf{x},\tilde{u}_0(\textbf{x})) -\alpha (\textbf{x},\lambda )|_{+} = 0, \quad \text {for a.e.}~\textbf{x}\in {{\mathbb {R}}}^d. \end{aligned}$$

Additionally, leveraging again the monotonicity of $\lambda \mapsto \alpha (\textbf{x},\lambda )$, we have

1.6 $$\begin{aligned} \begin{aligned} \max _{\lambda >1}|\alpha (\textbf{x},\tilde{u}_0(\textbf{x})) -\alpha (\textbf{x},\lambda )|_{+}&\le |\alpha (\textbf{x},\tilde{u}_0(\textbf{x})) -\alpha (\textbf{x},\min \{1,u_0(\textbf{x})\})|_{+}\\&\le |\alpha (\textbf{x},\tilde{u}_0(\textbf{x}))| + |\alpha (\textbf{x},\min \{1,u_0(\textbf{x})\})| \\&\le 2|\alpha (\textbf{x},\tilde{u}_0(\textbf{x}))| = 2|u_0(\textbf{x})|\in L^1(\mathbb {R}^d). \end{aligned} \end{aligned}$$

By combining (1.5) and (1.6), and applying the dominated convergence theorem, we conclude that $I_0^+(\lambda ) \rightarrow 0$ as $\lambda \rightarrow +\infty$. The analysis for $I_0^-$ is analogous.

Condition (i) is a generalization of (H3’) from [4] since, as shown in [14], (H3’) from [4] implies the existence of the functions *g* and $\beta$ such that ${\mathfrak {f}}(\textbf{x},u)=g(\beta (\textbf{x},u))$. In addition, we note that in [4, 14], the condition of boundedness of $\beta (\textbf{x},\lambda )$ by two continuous functions $h_{-}(\lambda )$ and $h_{+}(\lambda )$ is imposed. These functions have absolute values that tend to infinity and satisfy the inequality $h_{-}(\lambda ) \le |\beta (x,\lambda )| \le h_{+}(\lambda )$. We replace this condition with assumptions (i) (see (1.2)) and (ii). Specifically, we require *BV* regularity and boundedness of $\beta$ at the single point $\lambda =0$, alongside $L^1$-control of the $\textbf{x}$-variation of $\beta ^{-1}$. The growth conditions from [4, 14], as well as assumptions (1.2) and (ii) presented here, appear to lack a direct relation.

One significant difference between our work and previous contributions is that we allow initial data belonging only to $L^1({{\mathbb {R}}}^d)$. It is worth noting that a pure $L^1$ theory for conservation laws is not a new idea, and appropriate results can be found in [7, 9] (see also the references therein), but these works consider $\textbf{x}$-independent fluxes. In our case, we consider flux functions with non-smooth $\textbf{x}$-dependence.

From a different functional analytic perspective, as opposed to a purely PDE approach, the paper [1] considers a related subject. It presents a more general admissibility criterion but is limited to one-dimensional scenarios and a single-jump $\textbf{x}$-dependent flux. In this specific context, it provides an abstract method for formulating the problem with $L^1$ data, allowing for the direct application of the semi-group theory developed in [6, 10].

The problem (1.1) is an example of a conservation law with discontinuous flux. This type of equation has received a lot of attention since it represents a non-trivial generalization of scalar conservation laws [13], and it describes various physical processes in highly heterogeneous media. It is especially interesting because it generates several stable semi-groups of solutions. These solutions are mostly described in [2], but only in the case where the flux has a single discontinuity point. Most of the concepts described in [2] use uniqueness proofs based on the behavior of admissible solutions in a neighborhood of the interface (discontinuity point). A generalization of this approach to the case with multiple discontinuity points can be found in [3, 11].

As far as we are aware, the first uniqueness proof without considering the behavior around the interface has been proposed in [4] (see also [5]), but under more restrictive assumptions on the flux. The approach of [4] was generalized in [14], where it was also explained how to apply the method in the multi-dimensional case, precisely written later in [8]. It is worth noting that in [16], under conditions (H1), (H2), and (H3) from [4], the authors examined *BV* solutions to (1.1). For more recent developments in this area, we refer to [12] and its references.

The goal of the paper is to prove existence and uniqueness of appropriately defined solutions to (1.1). Let us now briefly explain the approach. We set

$$\begin{aligned} \tilde{u}=\beta (\textbf{x},u) \ \ \textrm{and} \ u:=\alpha (\textbf{x},\tilde{u})=:\beta ^{-1}(\textbf{x},\tilde{u}), \end{aligned}$$

and rewrite (1.1) as

$$\begin{aligned} \begin{aligned}&\partial _t u +{{\,\textrm{div}\,}}_\textbf{x}g(\beta (\textbf{x},u))=0, \quad u|_{t=0}=u_0 \in L^1({{\mathbb {R}}}), \end{aligned} \end{aligned}$$

and, with $\tilde{u}_0:= \beta (\textbf{x},u_0)$, as

$$\begin{aligned} \partial _t \alpha (\textbf{x},\tilde{u})+{{\,\textrm{div}\,}}_\textbf{x}g(\tilde{u})=0, \quad \alpha (\textbf{x},\tilde{u})|_{t=0}=\alpha (\textbf{x},\tilde{u}_0) \in L^1({{\mathbb {R}}}). \end{aligned}$$

To construct a solution, we need the following approximations:

$$\begin{aligned} \begin{aligned} \alpha _L(\textbf{x},\Lambda )&= {\left\{ \begin{array}{ll} \alpha (\textbf{x},-L) &  \Lambda \le -L, \\ \alpha (\textbf{x},\Lambda ), &  |\Lambda |< L, \\ \alpha (\textbf{x},L), &  \Lambda \ge L. \end{array}\right. } \\ \beta _L(\textbf{x},\lambda )&= {\left\{ \begin{array}{ll} -L, &  \lambda \le \alpha (\textbf{x},-L),\\ \alpha ^{-1}(\textbf{x},\lambda ) =\beta (\textbf{x},\lambda ), &  \lambda \in \bigl (\alpha (\textbf{x},-L),\alpha (\textbf{x},L)\bigr ),\\ L, &  \lambda \ge \alpha (\textbf{x},L), \end{array}\right. } \\ g_L(\Lambda )&= {\left\{ \begin{array}{ll} g(-L), &  \Lambda \le L, \\ g(\Lambda ), &  |\Lambda | < L, \\ g(L), &  \Lambda \ge L. \end{array}\right. } \end{aligned} \end{aligned}$$

The definition of $\beta _L$ for $\lambda \notin \bigl [\alpha (\textbf{x},-L),\alpha (\textbf{x},L)\bigr ]$ will not be important because, for the relevant solutions, the values of $\tilde{u}$ will be constrained to $[-L,L]$. We note that since $\alpha$, $\beta$, and *g* may not belong to $L^1({{\mathbb {R}}}^{d}\times {{\mathbb {R}}})$ or $L^1({{\mathbb {R}}})$, respectively, we cannot say anything about the convergence of the sequences $\Vert \alpha _L-\alpha \Vert _{L^1({{\mathbb {R}}}^{d}\times {{\mathbb {R}}})}$, $\Vert \beta _L-\beta \Vert _{L^1({{\mathbb {R}}}^{d}\times {{\mathbb {R}}})}$, or $\Vert g_L-g\Vert _{L^1({{\mathbb {R}}})}$.

We consider the following approximation problem:

1.7 $$\begin{aligned}&\partial _t u_n^L +{{\,\textrm{div}\,}}_\textbf{x}g_L(\beta _{L,n}(\textbf{x},u_n^L))= 0,\end{aligned}$$

1.8 $$\begin{aligned}&u_n^L|_{t=0}=u^L_0(\textbf{x}), \end{aligned}$$

where $u^L_0\in \bigl (L^1\cap L^\infty \bigr )({{\mathbb {R}}}^d)$ is an approximation of $u_0\in L^1({{\mathbb {R}}}^d)$ such that $u^L_0\rightarrow u_0$ in $L^1({\mathbb {R}}^d)$ as $L\rightarrow \infty$. Here, $\beta _{L,n}(\textbf{x},\lambda )$, for $n\in \mathbb {N}$, represents a smooth approximation of the function $\beta _L(\textbf{x},\lambda )$ in terms of the variable $\textbf{x}$. This approximation is achieved through a standard convolution process using a regularizing kernel. Note that this method of approximation preserves the monotonicity in $\lambda$. To be more precise,

$$\begin{aligned} \beta _{L,n}(\textbf{x},\lambda ) =\int _{{{\mathbb {R}}}} \beta _L(\textbf{y},\lambda )\,\omega _n(\textbf{x}-\textbf{y})\,d\textbf{y}, \end{aligned}$$

where $\omega _n$ is a standard mollifier satisfying

$$\begin{aligned} \omega _n(\textbf{z})\ge 0, \quad \int _{{{\mathbb {R}}}^d} \omega _n(\textbf{z})\,d\textbf{z}= 1, \quad \textrm{supp}\,\,\omega _n \subset B(0,1/n). \end{aligned}$$

Define

$$\begin{aligned} m_n(\textbf{x},-L):=\mathop {\mathrm {ess\,inf}}\limits _{|\textbf{x}-\textbf{y}|\le 1/n}\, \alpha (\textbf{y},-L), \qquad M_n(\textbf{x},L)=\mathop {\mathrm {ess\,sup}}_{|\textbf{x}-\textbf{y}|\le 1/n}\, \alpha (\textbf{y},L). \end{aligned}$$

Then we have

$$\begin{aligned} \beta _{L,n}(\textbf{x},\lambda ) = {\left\{ \begin{array}{ll} -L, &  \text {if } \lambda \le m_n(\textbf{x},-L),\\ \hbox { strictly increasing in}\ \lambda , &  \text {if } m_n(\textbf{x},-L)<\lambda <M_n(\textbf{x},L),\\ L, &  \text {if } \lambda \ge M_n(\textbf{x},L). \end{array}\right. } \end{aligned}$$

Note that $\bigl [m_n(\textbf{x},-L),M_n(\textbf{x},L)\bigr ] \supset \bigl [\alpha (\textbf{x},-L),\,\alpha (\textbf{x},L)\bigr ]$. Given that $\alpha (\cdot ,\pm L)$ are finite a.e., we have $m_n(\textbf{x},-L)\rightarrow \alpha (\textbf{x},-L)$ and $M_n(\textbf{x},L)\rightarrow \alpha (\textbf{x},L)$ as $n\rightarrow \infty$, for a.e. $\textbf{x}$. Furthermore, the semi-norm $|\beta _{L,n}(\cdot ,0)|_{BV({{\mathbb {R}}}^d)}$ remains uniformly bounded by $|\beta (\cdot ,0)|_{BV({{\mathbb {R}}}^d)}<\infty$ (we will make use of this fact later).

An inverse of $\beta _{L,n}$ with respect to $\lambda$, denoted by $\alpha _{L,n}$, is defined by

$$\begin{aligned} \alpha _{L,n}\bigl (\textbf{x},\Lambda \bigr ) = {\left\{ \begin{array}{ll} m_n(\textbf{x},-L), &  \text {if } \Lambda \le -L,\\ \bigl (\beta _{L,n}(\textbf{x},\cdot )\bigr )^{-1}\!(\Lambda ), &  \text {if } -L< \Lambda < L,\\ M_n(\textbf{x},L), &  \text {if } \Lambda \ge L. \end{array}\right. } \end{aligned}$$

On the open interval $(-L,L)$, the function $\beta _{L,n}(\textbf{x},\lambda )$ is strictly increasing in $\lambda$ for $\lambda \in \bigl (m_n(\textbf{x},-L),M_n(\textbf{x},L)\bigr )$, so there is a unique $\lambda$ solving $\beta _{L,n}(\textbf{x},\lambda )=\Lambda$. Hence one can write $\alpha _{L,n}(x,\Lambda ) =\bigl (\beta _{L,n}(x,\cdot )\bigr )^{-1}\!(\Lambda )$ for $-L<\Lambda <L$. At the flat zones where $\Lambda =-L$ or $\Lambda =L$, we set $\alpha _{L,n}(\textbf{x},\Lambda )$ to $m_n(\textbf{x},-L)$ or $M_n(\textbf{x},L)$, respectively, though any value in those plateau intervals would also be consistent. Our definition also sends all $\Lambda$ outside the range $(-L,L)$ to the boundary points $m_n(\textbf{x},-L)$ or $M_n(\textbf{x},L)$, aligning with the fact that $\beta _{L,n}(\textbf{x},\lambda )$ is constant ($-L$ or *L*) outside the interval $\bigl (m_n(\textbf{x},-L),M_n(\textbf{x},L))$ By definition of $m_n$, for a.e. $\textbf{y}$ with $|\textbf{x}-\textbf{y}|\le 1/n$, we have $m_n(\textbf{x},-L)\le \alpha (\textbf{y},-L)$, hence $\beta _L\bigl (\textbf{y},m_n(\textbf{x},-L)\bigr )=-L$ on that set of $\textbf{y}$. Thus $\beta _{L,n}\bigl (\textbf{x},m_n(\textbf{x},-L)\bigr ) =\int \beta _L\bigl (\textbf{y},m_n(\textbf{x},-L)\bigr ) \,\omega _n(\textbf{x}-\textbf{y})\,d\textbf{y}=-L$. A symmetric argument using shows that $\beta _{L,n}\bigl (\textbf{x},M_n(\textbf{x},L)\bigr )=L$. Summarizing, the following global composition identities hold for all real $\lambda$ and $\Lambda$:

$$\begin{aligned}&\alpha _{L,n}\bigl (\textbf{x}, \beta _{L,n}\bigl (\textbf{x},\lambda \bigr )\bigr ) = {\left\{ \begin{array}{ll} m_n(\textbf{x},-L), &  \text {if } \lambda \le m_n(\textbf{x},-L),\\ \lambda , &  \text {if } m_n(\textbf{x},-L)<\lambda<M_n(\textbf{x},L),\\ M_n(\textbf{x},L), &  \text {if } \lambda \ge M_n(\textbf{x},L). \end{array}\right. } \\  &\beta _{L,n}\bigl (\textbf{x}, \alpha _{L,n}\bigl (\textbf{x},\Lambda \bigr )\bigr ) = {\left\{ \begin{array}{ll} -L, &  \text {if } \Lambda \le -L,\\ \Lambda , &  \text {if } -L<\Lambda <L,\\ L, &  \text {if } \Lambda \ge L. \end{array}\right. } \end{aligned}$$

For every $n\in {{\mathbb {N}}}$, the problem (1.7), (1.8) admits a unique admissible solution $u_n^L$ in the sense of Kružkov, such that

1.9 $$\begin{aligned} u_n^L \in L^\infty ({{\mathbb {R}}}^+\times {{\mathbb {R}}}^d) \cap L^\infty ([0,T]; L^1({{\mathbb {R}}}^d)), \quad T>0. \end{aligned}$$

We note that, in general, the global property $u_n^L \in L^\infty ({{\mathbb {R}}}^+\times {{\mathbb {R}}}^d)$ does not necessarily hold. However, since the problem admits two stationary solutions $\alpha _{L,n}(\textbf{x},\pm L)$, and if we assume that $u_0^L$ (see (1.8)) satisfies

1.10 $$\begin{aligned} \alpha (\textbf{x},-L)\le \alpha _{L,n}(\textbf{x},-L)\le u^L_0(\textbf{x}) \le \alpha _{L,n}(\textbf{x},L)\le \alpha (\textbf{x},L), \quad \textbf{x}\in {{\mathbb {R}}}^d, \end{aligned}$$

the maximum principle ensures that $\alpha _{L,n}(\cdot ,-L)\le u_n^L(t,\cdot )\le \alpha (\cdot ,L)$ for all *t*. Let us be a bit more precise. Defining

$$\begin{aligned} \tilde{u}_n^L:=\beta _{L,n}(\textbf{x},u_n^L), \end{aligned}$$

and so $u_n^L=\alpha _{L,n}(\textbf{x},\tilde{u}_n^L)$, we obtain the equation

1.11 $$\begin{aligned} \partial _t \alpha _{L,n}(\textbf{x},\tilde{u}_n^L) +{{\,\textrm{div}\,}}_\textbf{x}g_L(\tilde{u}_n^L)=0, \end{aligned}$$

solutions of which satisfy the maximum principle:

$$\begin{aligned} \alpha _{L,n}(\textbf{x},-L) \le \alpha _{L,n}(\textbf{x},\tilde{u}_n^L)(t,\textbf{x}) \le \alpha _{L,n}(\textbf{x},L), \end{aligned}$$

recalling that $u_0^L=u_n^L(0,\textbf{x}) =\alpha _{L,n}(\textbf{x},\tilde{u}_n^L(0,\textbf{x}))$ is bounded below by $\alpha _{L,n}(\textbf{x},-L)$ and above by $\alpha _{L,n}(\textbf{x},L)$ (see (1.10)).

Next, we recall the Kružkov admissibility conditions for (1.7). For every $k\in {{\mathbb {R}}}$, the following inequality holds in the sense of distributions:

1.12 $$\begin{aligned} \begin{aligned} \partial _t |u_n^L-k|&+{{\,\textrm{div}\,}}_\textbf{x}\textrm{sgn}(u_n^L-k)\bigl (g_L(\beta _{L,n}(\textbf{x},u_n^L)) -g_L(\beta _{L,n}(\textbf{x},k))\bigr ) \\  &+ \textrm{sgn}(u_n^L-k){{\,\textrm{div}\,}}_\textbf{x}g_L(\beta _{L,n}(\textbf{x},k))\le 0. \end{aligned} \end{aligned}$$

Setting $k=0$ and testing the equation against a standard convolution kernel $\omega _\varepsilon =\frac{1}{\varepsilon ^d} \omega \bigl (\frac{\textbf{x}-\textbf{y}}{\varepsilon }\bigr )$, where $\textbf{y}\in {{\mathbb {R}}}^d$ is fixed, followed by integration over $\textbf{y}\in {{\mathbb {R}}}^d$, we obtain

$$\begin{aligned} \begin{aligned} \partial _t \int _{{{\mathbb {R}}}^d}|u_n^L(t,\cdot )|\star \omega _\varepsilon \, d\textbf{y}&\le \int _{{{\mathbb {R}}}^d} \left| \left( \textrm{sgn}(u_n^L) {{\,\textrm{div}\,}}_\textbf{x}g_L(\beta _{L,n}(\textbf{x},0))\right) \star \omega _\varepsilon \right| \, d\textbf{y}\\  &\le \Vert g_L\Vert _{\text {Lip}([-M,M])}|\beta _{L,n}(\cdot ,0)|_{BV({{\mathbb {R}}}^d)}, \end{aligned} \end{aligned}$$

where the last step follows from the Young convolution inequality, and $M:=\Vert \beta (\cdot ,0)\Vert _{L^\infty ({{\mathbb {R}}}^d)}$ (see the second part of (1.2)). To zero out the first divergence term in (1.12), we utilize the fact that the expression inside ${{\,\textrm{div}\,}}_\textbf{x}$ belongs to $L^1({{\mathbb {R}}}^d)$, uniformly over the interval $t \in [0,T]$ with $T>0$. This is supported by (1.9) and that $\Vert g_L\Vert _{\textrm{Lip}({{\mathbb {R}}})}\lesssim _{L} 1, \, \Vert \beta _{L,n}(\textbf{x},\cdot ) \Vert _{\textrm{Lip}({{\mathbb {R}}})} \lesssim _{L,n} 1$, for fixed *L* and *n*. Integrating over [0, *t*] and taking the limit $\varepsilon \rightarrow 0$, we obtain

1.13 $$\begin{aligned} \Vert u_n^L(t,\cdot )\Vert _{L^1({{\mathbb {R}}}^d)} \le \Vert u_0\Vert _{L^1({{\mathbb {R}}}^d)} +t \Vert g\Vert _{\text {Lip}([-M,M])}|\beta (\cdot ,0)|_{BV({{\mathbb {R}}}^d)}. \end{aligned}$$

The smoothness of $\alpha _{L,n}(\cdot ,\lambda )$ guarantees that $\alpha _{L,n}(\textbf{x},\lambda )$ is an admissible solution to (1.7) for every $\lambda \in {{\mathbb {R}}}$. Indeed, by taking $\tilde{u}_n^L=L$ in (1.11), we get

1.14 $$\begin{aligned} \partial _t \alpha _{L,n}(\textbf{x},\lambda )+{{\,\textrm{div}\,}}_\textbf{x}g_L(\lambda )=0. \end{aligned}$$

As Kružkov admissible solutions to the conservation law (1.7), the functions $u_n^L$ and $\alpha _{L,n}(\textbf{x},\lambda )$ adhere to the following comparison inequality for every $\lambda \in {{\mathbb {R}}}$:

$$\begin{aligned}&\partial _t |u_n^L-\alpha _{L,n}(\textbf{x},\lambda )|_\pm \\&\qquad \quad +{{\,\textrm{div}\,}}_{\textbf{x}} \bigl (\textrm{sgn}_\pm (u_n^L -\alpha _{L,n}(\textbf{x},\lambda )) (g_L(\beta _{L,n}(\textbf{x},u_n^L)) -g_L(\lambda ))\bigr ) \le 0. \end{aligned}$$

We can rewrite this inequality in the form

1.15 $$\begin{aligned}&\partial _t |\alpha ^L_n(\textbf{x},\tilde{u}_n^L)-\alpha ^L_n(\textbf{x},\lambda )|_\pm +{{\,\textrm{div}\,}}_{\textbf{x}} \left( \textrm{sgn}_\pm (\tilde{u}_n^L-\lambda ) (g_L(\tilde{u}_n^L)-g_L(\lambda ))\right) \le 0, \end{aligned}$$

where $$\alpha ^L_n(\textbf{x},\tilde{u}_n^L)=u_n^L$$.

To establish the existence of an admissible solution to (1.1), we will prove that the sequence $(u_n^L)$ converges to an admissible solution of (1.1) as $n\rightarrow \infty$ and then as $L\rightarrow \infty$. To do this, we will adapt an approach from [9, 15] by deriving kinetic equations for $\textrm{sgn}_{\pm }(\tilde{u}_n^L-\lambda )$ and taking limits as $n\rightarrow \infty$ and $L\rightarrow \infty$ along appropriate subsequences to define generalised kinetic functions $h_\pm (t,\textbf{x},\lambda )$. Then, we show that the integral of the product of $h_+(t,\textbf{x},\lambda )$ and $h_-(t,\textbf{x},\lambda )$ is non-decreasing over time, which implies that $h_+(t,\textbf{x},\lambda ) =\textrm{sgn}_+(\tilde{u}(t,\textbf{x})-\lambda )$ for some function $\tilde{u}$ representing the solution to (1.1).

We emphasize that the assumptions (i) and (ii) are sufficient but not necessarily optimal. The challenge is to establish the existence of a solution *u* with a finite $L^\infty ([0,T]; L^1({{\mathbb {R}}}^d))$ norm while ensuring that it meets the admissibility criteria that guarantee uniqueness. As noted by a referee, another approach to the $L^1$ bound is to assume the existence of a “particular solution” $u^\star \in L^\infty ([0,T]; L^1({{\mathbb {R}}}^d)) \cap L^\infty ([0,T] \times {{\mathbb {R}}}^d)$ to (1.1), satisfying the admissibility conditions from [4] or, more generally, from [14]. Then, applying the $L^1$ stability results from these works yields the bound

$$\begin{aligned} \begin{aligned} \Vert u_L\Vert _{L^\infty ([0,T]; L^1({{\mathbb {R}}}^d))}&\le \Vert u^\star \Vert _{L^\infty ([0,T]; L^1({{\mathbb {R}}}^d))} +\Vert u_L-u^\star \Vert _{L^\infty ([0,T]; L^1({{\mathbb {R}}}^d))} \\  &\le \Vert u^\star \Vert _{L^\infty ([0,T]; L^1({{\mathbb {R}}}^d))} +C\Vert u^L_0-u^\star (0,\cdot )\Vert _{L^1({{\mathbb {R}}}^d))}, \end{aligned} \end{aligned}$$

for any admissible solution $u_L$ corresponding to the initial data $u_0^L$. The right-hand side remains uniformly bounded as $L \rightarrow \infty$, thereby eliminating the need for the *BV* regularity assumption on $\beta (\textbf{x},0)$ in condition (i). A particularly interesting case of this approach is when $u^\star \equiv 0$, which occurs if $\beta (\textbf{x},0) \equiv 0$.

In Section 2, we provide a precise definition of admissible solutions to (1.1) and demonstrate their existence using the strategy just described. In Section 3, we prove a uniqueness result under an additional assumption on the flux.

## Existence of admissible solutions

In this section, we present the admissibility conditions and prove the existence of the corresponding solution. The solution concept is based on the approach outlined in [9]. We begin by introducing the following definition:

### Definition 1

We say that the function *u* is an admissible solution to (1.1) on [0, *T*], $T>0$, if $\tilde{u}=\beta (\textbf{x},u) \Longleftrightarrow u=\alpha (\textbf{x},\tilde{u})$ satisfies the following conditions:

(a) $$\alpha (\textbf{x},\tilde{u}(t,\textbf{x}))\in L^1([0,T]\times {{\mathbb {R}}}^d)$$;

(b) there exist non-negative measures $m^\pm _{\tilde{u}} \in {{\mathcal {M}}}([0,T]\times {{\mathbb {R}}}^d \times {{\mathbb {R}}})$ such that the following equation holds in ${{\mathcal {D}}}'((0,T)\times {{\mathbb {R}}}^d \times {{\mathbb {R}}})$:

$$\begin{aligned} \alpha '(\textbf{x},\lambda )\partial _t \, \textrm{sgn}_\pm (\tilde{u}(t,\textbf{x})-\lambda ) +{{\,\textrm{div}\,}}_{\textbf{x}} \big ( g'(\lambda ) \textrm{sgn}_\pm (\tilde{u}(t,\textbf{x})-\lambda )\big ) =\partial _\lambda m^\pm _{\tilde{u}}(t,\textbf{x},\lambda ), \end{aligned}$$

where (here and in the sequel) $\alpha '(\textbf{x},\lambda ):=\partial _\lambda \alpha (\textbf{x},\lambda )$;

(c) The following initial condition holds in the sense of weak traces:

$$\begin{aligned} \textrm{sgn}_\pm (\tilde{u}(t,\textbf{x})-\lambda )\big |_{t=0} =\textrm{sgn}_\pm (\tilde{u}_0(\textbf{x})-\lambda ), \quad \tilde{u}_0=\beta (x,u_0), \end{aligned}$$

i.e., for every $$\varphi \in C_c({{\mathbb {R}}}^d\times {{\mathbb {R}}})$$,

$$\begin{aligned} \lim \limits _{t\rightarrow 0}\iint \limits _{{{\mathbb {R}}}^d\times {{\mathbb {R}}}} \textrm{sgn}_\pm (\tilde{u}(t,\textbf{x})-\lambda )\varphi (\textbf{x},\lambda ) \, d\lambda d\textbf{x}=\iint \limits _{{{\mathbb {R}}}^d\times {{\mathbb {R}}}}\textrm{sgn}_\pm (\tilde{u}_0(\textbf{x})-\lambda ) \varphi (\textbf{x},\lambda ) \, d\lambda d\textbf{x}. \end{aligned}$$

(d) The measures $m^\pm _{\tilde{u}}$ satisfy the following decay property: for some bounded functions $\Gamma ^\pm _{\tilde{u}}:{{\mathbb {R}}}\rightarrow {{\mathbb {R}}}$ that vanishes at infinity,

2.1 $$\begin{aligned} \int _0^T\int _{{{\mathbb {R}}}^d} \, dm^\pm _{\tilde{u}}(t,\textbf{x},\lambda ) \le \Gamma ^\pm _{\tilde{u}}(\lambda ) \rightarrow 0 \ \ \textrm{as} \ \ \lambda \rightarrow \pm \infty . \end{aligned}$$

### Remark 2

For later reference, it is worth noting that when we define the functions

$$\begin{aligned}&h_+(\lambda ,u)=\textrm{sgn}_+(\tilde{u}-\lambda ) =\textbf{1}_{\lambda <u},\\&h_-(\lambda ,u)=h_+-1=-\textbf{1}_{\lambda \ge u} =\textrm{sgn}_-(\tilde{u}-\lambda ), \end{aligned}$$

for $\lambda ,u\in {{\mathbb {R}}}$, which both are non-increasing in $\lambda$ ($\partial _\lambda h_\pm \le 0$), we have the following relationships:

$$\begin{aligned} S(u)=\int _{{{\mathbb {R}}}} S'(\lambda )h_+(\lambda ,u)\, d\lambda , \quad u \in {{\mathbb {R}}}, \end{aligned}$$

for any locally Lipschitz continuous function $S:{{\mathbb {R}}}\rightarrow {{\mathbb {R}}}$ with $S\equiv 0$ on $(-\infty ,r]$ for some $r\in {{\mathbb {R}}}$, and

$$ S(u)=\int _{{{\mathbb {R}}}} S'(\lambda )h_-(\lambda ,u)\, d\lambda , \quad u \in {{\mathbb {R}}}, $$

if $S\equiv 0$ on $[r,\infty )$ for some $r\in {{\mathbb {R}}}$. Moreover, for any locally Lipschitz $S:{{\mathbb {R}}}\rightarrow {{\mathbb {R}}}$,

$$ \int -S'(\lambda )h_\pm (\lambda ,u)h_\mp (\lambda ,v)\, d\lambda =|S(u)-S(v)|_\pm , \quad u,v\in {{\mathbb {R}}}.$$

The following theorem is the central result of this paper.

### Theorem 3

(existence) Suppose conditions (i), (ii), and (iii) hold. There exists an admissible solution to (1.1) in the sense of Definition 1.

### Proof

It follows from (1.15) that

2.2 $$\begin{aligned} \begin{aligned} \partial _t |\alpha _{L,n}(\textbf{x},\tilde{u}_n^L)-\alpha _{L,n}(\textbf{x},\lambda ))|_\pm +{{\,\textrm{div}\,}}_{\textbf{x}} \left( \textrm{sgn}_\pm (\tilde{u}_n^L-\lambda ) (g_L(\tilde{u}_n^L)-g_L(\lambda ))\right)&\\ = -\tilde{m}^\pm _{n,L}(t,\textbf{x},\lambda ), \end{aligned} \end{aligned}$$

for some non-negative $\tilde{m}^\pm _{n,L}\in C({{\mathbb {R}}}_\lambda ;{{\mathcal {M}}}([0,T]\times {{\mathbb {R}}}^d)-w)$, i.e., the functionals $\tilde{m}^\pm _{n,L}$ are continuous in $\lambda$ with respect to the weak-$\star$ topology of ${{\mathcal {M}}}$. We use ${{\mathcal {M}}}([0,T]\times {{\mathbb {R}}}^d)$ to denote the space of Radon measures over $[0,T]\times {{\mathbb {R}}}^d$.

Integrating (1.15) over $[0,t]\times {{\mathbb {R}}}^d$ yields

$$\int _{{{\mathbb {R}}}^d}|\alpha _{L,n}(\textbf{x},\tilde{u}_n^L(t,\textbf{x})) -\alpha _{L,n}(\textbf{x},\lambda )|_\pm \, d\textbf{x}\le \int _{{{\mathbb {R}}}^d}|\alpha _{L,n}(\textbf{x},\tilde{u}_0(\textbf{x})) -\alpha _{L,n}(\textbf{x},\lambda )|_\pm \, d\textbf{x}.$$

Using this, it follows from (2.2) that

2.3 $$\begin{aligned} \int _0^T\int _{{{\mathbb {R}}}^d} \, d\tilde{m}^\pm _{n,L}(t,\textbf{x},\lambda ) \le \int _{{{\mathbb {R}}}^d}|\alpha _{L,n}(\textbf{x},\tilde{u}_0(\textbf{x})) -\alpha _{L,n}(\textbf{x},\lambda )|_\pm \, d\textbf{x}:={\tilde{\Gamma }}_{n,L}^\pm (\lambda ).\nonumber \\ \end{aligned}$$

Moreover,

2.4 $$\begin{aligned} \lim \limits _{L,n\rightarrow \infty } {\tilde{\Gamma }}_{n,L}^\pm (\lambda ) =\int _{{{\mathbb {R}}}^d}|\alpha (\textbf{x},\tilde{u}_0(\textbf{x})) -\alpha (\textbf{x},\lambda )|_\pm \, d\textbf{x}, \end{aligned}$$

where, according to (1.4), the right-hand side tends to zero as $\lambda \rightarrow \pm \infty$.

Next, we compute the derivative of (2.2) with respect to $\lambda$, yielding

2.5 $$\begin{aligned} \alpha _{L,n}'(\textbf{x},\lambda ) \partial _t h_{\pm ,L,n} +{{\,\textrm{div}\,}}_{\textbf{x}}\left( g'_L(\lambda ) h_{\pm ,L,n}\right) =\partial _{\lambda } \tilde{m}^\pm _{n,L}(t,\textbf{x},\lambda ), \end{aligned}$$

where $$\alpha _{L,n}'(\textbf{x},\lambda ) :=\partial _\lambda \alpha _{L,n}(\textbf{x},\lambda )$$ and

2.6 $$\begin{aligned} h_{\pm ,L,n}=h_{\pm ,L,n}(t,\textbf{x},\lambda ) :=\textrm{sgn}_\pm (\tilde{u}_n^L(t,\textbf{x})-\lambda ). \end{aligned}$$

Since the sequences $(h_{\pm ,L,n})_{n\in {{\mathbb {N}}}}$ are bounded, there are subsequences (not relabelled) that converge weakly-$\star$ in $L^\infty$ to limits $h_{\pm ,L}$ as $n\rightarrow \infty$:

$$\begin{aligned} h_{\pm ,L,n} \overset{n\uparrow \infty }{\longrightarrow } h_{\pm ,L} \ \ \text {weakly- }\, \star \, \text { in} \ \ L^\infty ((0,T)\times {{\mathbb {R}}}^d\times {{\mathbb {R}}}). \end{aligned}$$

Similarly, there exist subsequences of $(\tilde{m}^\pm _{n,L})_{n\in {{\mathbb {N}}}}$, which we do not relabel, that converge weakly-$\star$ in ${{\mathcal {M}}}((0,T)\times {{\mathbb {R}}}\times {{\mathbb {R}}}^d)$ to some nonnegative limit measures $\tilde{m}^\pm _L$. By taking these subsequences in (2.5) for both $(h_{\pm ,L,n})_{n\in {{\mathbb {N}}}}$, $(\tilde{m}^\pm _{n,L})_{n\in {{\mathbb {N}}}}$ and letting $n\rightarrow \infty$, we obtain the following equations that hold in the sense of distributions:

2.7 $$\begin{aligned} \alpha _L'(\textbf{x},\lambda ) \partial _t h_{\pm ,L} +{{\,\textrm{div}\,}}_{\textbf{x}} \bigl (g'_L(\lambda ) h_{\pm ,L}\bigr ) = \partial _{\lambda } \tilde{m}^\pm _L(t,\textbf{x},\lambda ). \end{aligned}$$

We note that $h_{\pm ,L}$ do not have to be sign functions, but they must satisfy the properties of being non-increasing with respect to $\lambda$ and being between 0 and 1. This is sufficient for our purposes.

Now we take the convolution of (2.7) with respect to all the variables $(t,x,\lambda )$. To this end, introduce the rescaled mollifier

2.8 $$\begin{aligned} \omega _{\sigma ,\epsilon ,\eta }(t,\textbf{x},\lambda ) :=\frac{1}{\sigma \epsilon ^d \eta } \omega \left( \frac{t}{\sigma }\right) \prod _{k=1}^d \omega \left( \frac{x_k}{\epsilon }\right) \omega \left( \frac{\lambda }{\eta }\right) , \end{aligned}$$

where $\omega :{{\mathbb {R}}}\rightarrow {{\mathbb {R}}}$ is a non-negative, compactly supported function with unit mass. We need notation for the mollifications of the functions $h_{\pm ,L}$:

$$\begin{aligned}&h^{\sigma ,\epsilon ,\eta }_{\pm ,L}(t,\textbf{x},\lambda ) :=h_{\pm ,L}\star \omega _{\sigma ,\epsilon ,\eta }(t,\textbf{x},\lambda ). \end{aligned}$$

By testing the “$+$” equation in (2.7) with $\omega _{\sigma ,\epsilon ,\eta }$, we can derive the following equation in a standard manner:

2.9 $$\begin{aligned} \partial _t \left( \alpha '_L(\cdot _\textbf{x},\cdot _\lambda ) h_{+,L}\right) \star \omega _{\sigma ,\epsilon ,\eta } +{{\,\textrm{div}\,}}_\textbf{x}\bigl (g_L'(\cdot _\lambda ) h_{+,L} \bigr ) \star \omega _{\sigma ,\epsilon ,\eta } =\partial _\lambda \tilde{m}^+_L\star \omega _{\sigma ,\epsilon ,\eta }. \end{aligned}$$

We then multiply this equation by

$$\begin{aligned} h_{-,L}^{\sigma ,\epsilon ,\eta } =h_{-,L}\star \omega _{\sigma ,\epsilon ,\eta } (t,\textbf{x},\lambda ) =h_{+,L}^{\sigma ,\epsilon ,\eta }(t,\textbf{x},\lambda )-1 \end{aligned}$$

and integrate the resulting equation with respect to $\lambda \in {{\mathbb {R}}}$.

Since $\tilde{u}_n^L$ is bounded between $-L$ and *L*, the function $h_{+,L,n}(t,\textbf{x},\lambda )$ (see (2.6)) is supported on the interval $(-\infty , L)$, uniformly in *n*. Similarly, the function $h_{-,L,n}(t,\textbf{x},\lambda )$ is supported on the interval $(-L, \infty )$, also independently of *n*. As a result, the limits of these functions, $h_{+,L}$ and $h_{-,L}$, have the same support intervals. The product of these two functions is thus a $\lambda$-compactly supported function. This, along with the properties of the functions $\alpha '_L$ and $g'_L$, allows us to apply integration by parts (in $\lambda$) to arrive at the inequality

2.10 $$\begin{aligned} \begin{aligned}&\int _{{{\mathbb {R}}}} {h}_{-,L}^{\sigma ,\epsilon ,\eta } \partial _t\bigl ( \alpha _L' h_{+,L}\bigr ) \star \omega _{\sigma ,\epsilon ,\eta } \, d\lambda + \int _{{{\mathbb {R}}}}{h}_{-,L}^{\sigma ,\epsilon ,\eta } {{\,\textrm{div}\,}}_\textbf{x}\bigl (g_L' \, h_{+,L} \bigr ) \star \omega _{\sigma ,\epsilon ,\eta } \, d\lambda \ge 0, \end{aligned} \end{aligned}$$

where we have used the fact that $\tilde{m}^+_L \star \omega _{\sigma ,\epsilon ,\eta } \ge 0$ and that ${h}_{-,L}^{\sigma ,\epsilon ,\eta }$ is non-increasing in $\lambda$. By reversing the roles of ${h}_{-,L}^{\sigma ,\epsilon ,\eta }$ and ${h}_{+,L}^{\sigma ,\epsilon ,\eta }$ (more precisely, using the “−" equation in (2.7), multiplying by $h_{+,L}^{\sigma ,\epsilon ,\eta }$ and adjusting the subsequent steps accordingly), we obtain the inequality:

2.11 $$\begin{aligned} \begin{aligned}&\int _{{{\mathbb {R}}}}{h}_{+,L}^{\sigma ,\epsilon ,\eta } \partial _t\bigl ( \alpha _L' {h}_{-,L} \bigr ) \star \omega _{\sigma ,\epsilon ,\eta } \, d\lambda + \int _{{{\mathbb {R}}}}{h}_{+,L}^{\sigma ,\epsilon ,\eta } {{\,\textrm{div}\,}}_\textbf{x}\bigl (g_L'\, {h}_{-,L}\bigr ) \star \omega _{\sigma ,\epsilon ,\eta }\, d\lambda \ge 0. \end{aligned}\qquad \end{aligned}$$

We obtain the final result by adding together inequalities (2.10) and (2.11), and then taking the limit as $\eta \rightarrow 0$ (vanishing mollification in the velocity variable):

2.12 $$\begin{aligned} \begin{aligned}&\int _{{{\mathbb {R}}}} {h}_{+,L}^{\sigma ,\epsilon } \partial _t\bigl ( \alpha _L'(\cdot ,\lambda ) {h}_{-,L} \bigr ) \star \omega _{\sigma ,\epsilon }+{h}_{-,L}^{\sigma ,\epsilon } \partial _t\bigl ( \alpha _L'(\cdot ,\lambda ) h_{+,L}\bigr ) \star \omega _{\sigma ,\epsilon }\, d\lambda \\  &\qquad +\int _{{{\mathbb {R}}}} {h}_{+,L}^{\sigma ,\epsilon } {{\,\textrm{div}\,}}_\textbf{x}\bigl ( g_L'(\lambda ) {h}_{-,L}^{\sigma ,\epsilon } \bigr ) +h_{-,L}^{\sigma ,\epsilon } {{\,\textrm{div}\,}}_\textbf{x}\bigl ( g_L'(\lambda ) h^{\sigma ,\epsilon }_{+,L} \bigr )\, d\lambda \ge 0. \end{aligned} \end{aligned}$$

We may rewrite the inequality (2.12) in the form

2.13 $$\begin{aligned} \begin{aligned}&\int _{{{\mathbb {R}}}}\alpha _L'(\textbf{x},\lambda ) \partial _t \bigl ( {h}_{+,L}^{\sigma ,\epsilon } {h}^{\sigma ,\epsilon } _{-,L}\bigr ) \, d\lambda \\&\qquad +\int _{{{\mathbb {R}}}} {h}_{-,L}^{\sigma ,\epsilon } \partial _t\bigl ( \alpha _L'(\cdot _\textbf{x},\lambda ) h_{+,L}\bigr ) \star \omega _{\sigma ,\epsilon } -\alpha _L'(\textbf{x},\lambda ) h_{-,L}^{\sigma ,\epsilon } \partial _t h^{\sigma ,\epsilon }_{+,L}\, d\lambda \\&\qquad + \int _{{{\mathbb {R}}}} {h}_{+,L}^{\sigma ,\epsilon } \partial _t\bigl (\alpha _L'(\cdot _\textbf{x},\lambda ) h_{-,L}\bigr ) \star \omega _{\sigma ,\epsilon }- \alpha _L'(\textbf{x},\lambda ) h_{+,L}^{\sigma ,\epsilon }\partial _t {h}^{\sigma ,\epsilon }_{-,L} \, d\lambda \\&\qquad + \int _{{{\mathbb {R}}}} {{\,\textrm{div}\,}}_\textbf{x}\bigl ( g_L'(\lambda ) h_{-,L}^{\sigma ,\epsilon } {h}_{+,L}^{\sigma ,\epsilon } \bigr ) \, d\lambda \ge 0. \end{aligned} \end{aligned}$$

We will now examine the second term of (2.13). According to the definition of convolution, for any positive value of *T*, we have:

$$\begin{aligned} \begin{aligned}&\Big | \int _0^T\int _{{{\mathbb {R}}}^d}\int _{{{\mathbb {R}}}} {h}_{-,L}^{\sigma ,\epsilon } \partial _t\bigl (\alpha _L'(\cdot _\textbf{x},\lambda ) h_{+,L} \bigr ) \star \omega _{\sigma ,\epsilon } -\alpha _L'(\textbf{x},\lambda ) {h}_{-,L}^{\sigma ,\epsilon } \partial _t h^{\sigma ,\epsilon }_{+,L} \, d\lambda d\textbf{x}dt \Big | \\  &\quad \le \Big | \int _0^T\int _{{{\mathbb {R}}}^d}\int _{{{\mathbb {R}}}} \Big |\int _0^T\int _{{{\mathbb {R}}}^d} \frac{1}{\sigma ^2}\omega '\left( \frac{t-\tau }{\sigma }\right) \frac{1}{\epsilon ^d} \omega _x\left( \frac{\textbf{x}-\textbf{z}}{\epsilon }\right) \\  &\quad \qquad \qquad \qquad \qquad \qquad \times \bigl (\alpha _L'(\textbf{z},\lambda ) -\alpha _L'(\textbf{x},\lambda ) \bigr ) h^{\sigma ,\epsilon }_{+,L}(\tau ,\textbf{z},\lambda ) \, d\tau d\textbf{z}\Big | \, d\lambda d\textbf{x}dt\Big | \\  &\quad \le C \int _{{{\mathbb {R}}}^d} \int _{{{\mathbb {R}}}} \int _{{{\mathbb {R}}}^d} \frac{|\alpha _L'(\textbf{x}+\epsilon \textbf{y},\lambda ) -\alpha _L'(\textbf{x},\lambda )|}{\sigma } \omega _x(\textbf{y})\, d\textbf{y}d\textbf{x}d\lambda \\  &\quad \le \frac{C}{\sigma } \sup _{|\textbf{z}|\le \epsilon } \Vert \alpha _L'(\cdot +\textbf{z},\cdot )-\alpha _L'(\cdot ,\cdot ) \Vert _{L^1({{\mathbb {R}}}^d\times {{\mathbb {R}}})}, \end{aligned} \end{aligned}$$

where $\omega _x\left( \frac{\textbf{x}}{\epsilon }\right) := \prod _{k=1}^d\omega \left( \frac{x_k}{\epsilon }\right)$. The same estimate holds for the third term of (2.13). Next, note that

$$\begin{aligned} \alpha _L'(\textbf{x},\lambda )&= {\left\{ \begin{array}{ll} \alpha '(\textbf{x},\lambda ), &  |\lambda | < L, \\ 0, &  |\lambda |>L, \end{array}\right. } \end{aligned}$$

from which it follows that, for $|\textbf{z}|\le \epsilon$,

$$\begin{aligned} \begin{aligned}&\Vert \alpha _L'(\cdot +\textbf{z},\cdot )-\alpha _L'(\cdot ,\cdot ) \Vert _{L^1({{\mathbb {R}}}^d\times {{\mathbb {R}}})} \le \int _{-L}^L\int _{{{\mathbb {R}}}^d} |\alpha '(\textbf{x}+\textbf{z},\lambda )-\alpha '(\textbf{x},\lambda )| \, d\textbf{x}d\lambda \\  &\qquad \quad \overset{(3.1)}{\le } \rho (\epsilon )\int _{-L}^Lp(|\lambda |)\, d\lambda =:\varrho (\epsilon ){\tilde{p}}(L)\rightarrow 0, \quad \hbox { as}\ \epsilon \rightarrow 0, \end{aligned} \end{aligned}$$

for every fixed *L*. Although ${\tilde{p}}(L) \rightarrow \infty$ as $L\rightarrow \infty$, we may select $L=L(\epsilon )$ such that $L(\epsilon )\rightarrow \infty$ and simultaneously $\varrho (\epsilon ){\tilde{p}}(L(\epsilon )) \rightarrow 0$ as $\varepsilon \rightarrow 0$. Therefore

2.14 $$\begin{aligned} \begin{aligned}&\lim \limits _{\epsilon \rightarrow 0}\Big | \int _0^T\int _{{{\mathbb {R}}}^d}\int _{{{\mathbb {R}}}} \Big ( {h}_{-,L(\epsilon )}^{\sigma ,\epsilon } \, \partial _t\left( \alpha _{L(\epsilon )}'(\cdot _\textbf{x},\lambda ) h_{+,L(\epsilon )}\right) \star \omega _{\sigma ,\epsilon } \\  &\qquad \qquad \qquad \qquad \qquad -\alpha _{L(\epsilon )}'(\textbf{x},\lambda ) h_{-,L(\epsilon )}^{\sigma ,\epsilon } \, \partial _t h^{\sigma ,\epsilon }_{+,L(\epsilon )} \Big )\, d\lambda d\textbf{x}dt \Big |=0. \end{aligned} \end{aligned}$$

With (2.14) in mind, we can integrate equation (2.13) over the region $[0,t]\times {{\mathbb {R}}}^d$, and then take the limits as $\epsilon$ approaches 0 and $\sigma$ approaches 0 to obtain

$$\begin{aligned} \begin{aligned}&\lim \limits _{\sigma \rightarrow 0}\lim \limits _{\varepsilon \rightarrow 0} \int _{{{\mathbb {R}}}} \alpha _{L(\epsilon )}'(\textbf{x},\lambda ) {h}_{+,L(\epsilon )}^{\sigma ,\epsilon }(t,\textbf{x},\lambda ) {h}^{\sigma ,\epsilon } _{-,L(\epsilon )}(t,\textbf{x},\lambda )\, d\lambda \\&\qquad \ge \int _{{{\mathbb {R}}}}\alpha '(\textbf{x},\lambda ) \textrm{sgn}_+(\tilde{u}_0(\textbf{x})-\lambda ) \textrm{sgn}_-(\tilde{u}_0(\textbf{x})-\lambda ) \,d\lambda =0, \end{aligned} \end{aligned}$$

for almost every $(t,\textbf{x})$. Since $\alpha _{L(\epsilon )}'(\textbf{x},\lambda )>0$ for all $(\textbf{x},\lambda )\in {{\mathbb {R}}}^d\times (-L,L)$ for every fixed $L>0$, and $h_{+,L(\epsilon )}^{\sigma ,\epsilon } h^{\sigma ,\epsilon } _{-,L(\epsilon )}\le 0$ a.e., we conclude that for a.e. $(t, \textbf{x},\lambda )$,

2.15 $$\begin{aligned} \begin{aligned}&\lim \limits _{k\rightarrow \infty }\lim \limits _{n\rightarrow \infty } h_{+,L(\epsilon _n)}^{\sigma _k,\epsilon _n}(t, \textbf{x},\lambda ) h^{\sigma _k,\epsilon _n} _{-,L(\epsilon _n)}(t, \textbf{x},\lambda ) \\  &\qquad =\lim \limits _{k\rightarrow \infty }\lim \limits _{n\rightarrow \infty } \Big (\big (h^{\sigma _k,\epsilon _n}_{+,L(\epsilon _n)} (t, \textbf{x},\lambda )\big )^2 -h_{+,L(\epsilon _n)}^{\sigma _k,\epsilon _n} (t, \textbf{x},\lambda ) \Big )=0, \end{aligned} \end{aligned}$$

where the limits are taken along the subsequences $(\sigma _k)$ and $(\epsilon _n)$.

Now, we can take a (non-relabeled) subsequence of $({h}_{+,L(\epsilon _n)}^{\sigma _k,\epsilon _n})_{k,n}$ that weakly-$\star$ converges in $L^\infty ({{\mathbb {R}}}^+\times {{\mathbb {R}}}^{d+1})$ to a limit $h_+\in L^\infty ({{\mathbb {R}}}^+\times {{\mathbb {R}}}^{d+1})$ as $n\rightarrow \infty$ and then $k\rightarrow \infty$. It is worth noting that equation (2.15) implies that the sequence $\big (({h}_{+,L(\epsilon _n)}^{\sigma _k,\epsilon _n})^2 \big )_{\sigma _k,\epsilon _n}$ has the same weak-$\star$ limit. Thus, given any function $\varphi \in L^1$,

$$\begin{aligned} \lim \limits _{\sigma _k\rightarrow 0}\lim \limits _{\epsilon _n\rightarrow 0} \iiint \Bigl (h_{+,L(\epsilon _n)}^{\sigma _k,\epsilon _n}(t, \textbf{x},\lambda ) -h_+(t, \textbf{x},\lambda )\Bigr )^2 \varphi (t,\textbf{x},\lambda ) \, d\lambda dx dt=0, \end{aligned}$$

and thus, along non-relabeled subsequences $(\sigma _k)$ and $(\epsilon _n)$,

$$\begin{aligned} \lim \limits _{\sigma _k\rightarrow 0}\lim \limits _{\epsilon _n\rightarrow 0} h_{+,L(\epsilon _k)}^{\sigma _n,\epsilon _k}(t, \textbf{x},\lambda ) =h_+(t,\textbf{x},\lambda ) \quad \text {for a.e.} ~(t,\textbf{x},\lambda ). \end{aligned}$$

Hence, equation (2.15) leads to the conclusion that $h_+$ can only be either 0 or 1 almost everywhere. Additionally, since $h_+$ nonincreases as $\lambda$ increases, we can conclude that there exists a function $\tilde{u}:[0,T]\times {{\mathbb {R}}}^d \rightarrow {{\mathbb {R}}}$ such that

2.16 $$\begin{aligned} h_+(t,\textbf{x},\lambda )=\textrm{sgn}_+(\tilde{u}(t,\textbf{x})-\lambda ). \end{aligned}$$

We shall show that the function $\tilde{u}$ represents an admissible solution to (1.1) in the sense of Definition 1.

By choosing $L=L(\epsilon )$ in (2.9) in such a way that (2.16) holds, we can take the limits $\eta \rightarrow 0$, $\epsilon \rightarrow 0$, and $\sigma \rightarrow 0$ along the corresponding subsequences and obtain, in the sense of distributions,

2.17 $$\begin{aligned} \partial _t \left( \alpha '(\textbf{x},\lambda ) \, \textrm{sgn}_+(\tilde{u}(t,\textbf{x})-\lambda )\right) +{{\,\textrm{div}\,}}_\textbf{x}\left( g'(\lambda ) \, \textrm{sgn}_+(\tilde{u}(t,\textbf{x})-\lambda ) \right) =\partial _\lambda {\tilde{m}}_{\tilde{u}}^+,\qquad \end{aligned}$$

for a measure ${\tilde{m}}_{\tilde{u}}^+\ge 0$. A similar equation holds for

$$ h_-:=h_+-1 =\textrm{sgn}_-(\tilde{u}(t,\textbf{x})-\lambda ) $$

and a corresponding measure ${\tilde{m}}_{\tilde{u}}^-$. We observe that the decay property (2.1) for ${\tilde{m}}_{\tilde{u}}^\pm$ is satisfied according to (2.4).

To conclude, we now demonstrate that the initial condition is satisfied:

2.18 $$\begin{aligned} \textrm{sgn}_\pm (\tilde{u}(t,\textbf{x})-\lambda ) \overset{t\downarrow 0}{\longrightarrow } \textrm{sgn}_\pm (\tilde{u}_0(\textbf{x})-\lambda ) \ \ \textrm{in} \ \ {{\mathcal {D}}}'({{\mathbb {R}}}^d\times {{\mathbb {R}}}). \end{aligned}$$

It is sufficient to establish this for $\textrm{sgn}_+(\tilde{u}(t,\textbf{x})-\lambda )$. We first observe that condition (d) is satisfied due to (2.3) and (2.4). With this knowledge, we can repeat the procedure from [9, Proposition 5.1]. To do this, we consider the function

$$\varphi _\varepsilon (t) ={\left\{ \begin{array}{ll} \frac{\varepsilon -t}{\varepsilon }, &  0\le t \le \varepsilon \\ 0,&  t\ge \varepsilon \end{array}\right. }$$

and test (2.17) with $\varphi _\varepsilon (t)\phi (\textbf{x}) \rho (\lambda )$, $\phi \in C^1_c({{\mathbb {R}}}^d)$, $\rho \in C^1_c({{\mathbb {R}}})$. The result is

2.19 $$\begin{aligned} \begin{aligned}&\frac{1}{\varepsilon } \int _0^\varepsilon \iint _{{{\mathbb {R}}}^d\times {{\mathbb {R}}}} \alpha '(\textbf{x},\lambda ) \, \textrm{sgn}_+(\tilde{u}(t,\textbf{x})-\lambda )\phi (\textbf{x}) \rho (\lambda )\, dt d\textbf{x}d\lambda \\&\quad -\iint _{{{\mathbb {R}}}^d\times {{\mathbb {R}}}} \alpha '(\textbf{x},\lambda ) \, \textrm{sgn}_+(\tilde{u}_0(\textbf{x})-\lambda )\phi (\textbf{x}) \rho (\lambda ) \, d\textbf{x}d\lambda \\  &\quad +\int _0^\varepsilon \int _{{{\mathbb {R}}}^d\times {{\mathbb {R}}}} g'(\lambda ) \, \textrm{sgn}_+(\tilde{u}(t,\textbf{x})-\lambda ) \cdot \nabla \phi \, \rho (\lambda ) \varphi _\varepsilon (t) \, d\textbf{x}d\lambda dt\\&\quad \quad =-\int _0^\varepsilon \iint _{{{\mathbb {R}}}^d\times {{\mathbb {R}}}} \varphi _\varepsilon (t) \phi (\textbf{x}) \rho '(\lambda ) \, d m_{\tilde{u}}^+. \end{aligned} \end{aligned}$$

Let $\bar{h}^+(\textbf{x},\lambda )$ and $\bar{m}_{\tilde{u}}^+(\textbf{x},\lambda )$ denote, respectively, the $L^\infty ({{\mathbb {R}}}^d\times {{\mathbb {R}}})$ weak-$\star$ limit of $\frac{1}{\varepsilon } \int _0^\varepsilon \textrm{sgn}_+(\tilde{u}(t,\textbf{x})-\lambda )\,dt$ and the weak-$\star$ limit of $\int _0^\varepsilon \int _{{{\mathbb {R}}}^d\times {{\mathbb {R}}}} \varphi _\varepsilon (t) \, d m_{\tilde{u}}^+(t,\textbf{x},\lambda )$ in the space ${{\mathcal {M}}}({{\mathbb {R}}}^d\times {{\mathbb {R}}})$, as $\varepsilon \rightarrow 0$ along a common subsequence. Then, by letting $\varepsilon \rightarrow 0$ along this subsequence in equation (2.19), we arrive at

2.20 $$\begin{aligned} \partial _\lambda \bar{m}_{\tilde{u}}^+(\textbf{x},\lambda ) =\alpha '(\textbf{x},\lambda ) \left( H^+(\textbf{x},\lambda ) -\chi ^+(\lambda ;\tilde{u}_0(\textbf{x}))\right) , \end{aligned}$$

where $H^+(\textbf{x},\lambda ):= \bar{h}^+(\textbf{x},\lambda )-\textbf{1}_{\lambda <0}$ and $\chi ^+(\lambda ;\tilde{u}_0(\textbf{x})) :=\textrm{sgn}_+(\tilde{u}_0(\textbf{x})-\lambda ) -\textbf{1}_{\lambda <0}$. Since $\alpha '>0$, we conclude the proof of (2.18) using (2.20) as in [9, Proposition 5.1].

It remains to prove that $\alpha (\textbf{x},\tilde{u}(t,\textbf{x}))\in L^1({{\mathbb {R}}}^d_\textbf{x}\times {{\mathbb {R}}}_\lambda )$, for a.e. $t\in [0,T]$. We have proved that (along the previously chosen subsequences)

$$ \left( \alpha _{L(\varepsilon )}'(\cdot ,\lambda ) \textrm{sgn}_+(\tilde{u}^{L(\varepsilon )}_{n}-\lambda ) \right) \star \omega _{\sigma ,\varepsilon }(t,\textbf{x}) \overset{\sigma ,\varepsilon ,1/n\downarrow 0}{\longrightarrow } \alpha '(\textbf{x},\lambda )\,\textrm{sgn}_+(\tilde{u}(t,\textbf{x})-\lambda ), $$

almost everywhere. Thus, by the Fatou lemma,

$$\begin{aligned} \begin{aligned}&\int _{{{\mathbb {R}}}^d} |\alpha (\textbf{x},\tilde{u}(t,\textbf{x}))|_+ \, d\textbf{x}= \int _{{{\mathbb {R}}}^d} \int _{{{\mathbb {R}}}} \alpha '(\textbf{x},\lambda ) \textrm{sgn}_+(\tilde{u}(t,\textbf{x})-\lambda )\, d\lambda d\textbf{x}\\  &\quad \le \liminf \limits _{\sigma ,\varepsilon ,1/n\rightarrow 0} \int _{{{\mathbb {R}}}^d} \int _{{{\mathbb {R}}}} \left( \alpha _{L(\varepsilon )}'(\cdot ,\lambda ) \textrm{sgn}_+(\tilde{u}^{L(\varepsilon )}_{n}-\lambda )\right) \star \omega _{\sigma ,\varepsilon }(t,\textbf{x}) \, d\lambda d\textbf{x}\\&\quad =\liminf \limits _{\sigma ,\varepsilon ,1/n\rightarrow 0} \int _{{{\mathbb {R}}}^d} |\alpha _{L(\varepsilon )}(\cdot ,\tilde{u}^{L(\varepsilon )}_{n})|_+ \star \omega _{\sigma ,\varepsilon }(t,\textbf{x})\, d\textbf{x}\overset{(1.13)}{\le } C. \end{aligned} \end{aligned}$$

In the same way, we obtain $\int _{{{\mathbb {R}}}^d} |\alpha (\textbf{x},\tilde{u}(t,\textbf{x}))|_- \,d\textbf{x}\le C$. $\square$

## Uniqueness of admissible solutions

In this section, we will demonstrate the uniqueness of admissible solutions to the conservation law (1.1) under an additional Lipschitz condition on the flux.

### Theorem 4

(uniqueness) In addition to conditions (i) through (iii), assume that the following condition is also satisfied (here ${\mathfrak {f}}':=\partial _\lambda {\mathfrak {f}}'$):

(iv) $\exists \tilde{R}>0$ such that $|{\mathfrak {f}}'(\textbf{x},\lambda )| \le \tilde{R}$, for all $(\textbf{x},\lambda ) \in {{\mathbb {R}}}^d\times {{\mathbb {R}}}$.

Then, based on Definition 1, the admissible solution to (1.1) is unique.

### Remark 5

If the entropy solutions are bounded, it is sufficient to assume that (iv) holds for $\lambda \in [-M,M]$, where *M* is the bound on the solutions. Further information can be found in [8, 14].

### Proof

In the course of the proof, we will use the notation from the previous section, including the convolution kernels introduced there.

Let $u,\textsf{u}$ be two admissible solutions of (1.1) with initial data $u_0,\textsf{u}_0$, respectively. We know that the following two equations hold in the sense of distributions:

$$\begin{aligned}&\alpha '(\textbf{x},\lambda )\partial _t \, \textrm{sgn}_+(\tilde{u}(t,\textbf{x})-\lambda ) +{{\,\textrm{div}\,}}_{\textbf{x}} \big ( g'(\lambda ) \textrm{sgn}_+(\tilde{u}(t,\textbf{x})-\lambda )\big ) =\partial _\lambda m^+_{\tilde{u}}(t,\textbf{x},\lambda ),\\&\alpha '(\textbf{x},\lambda )\partial _t \, \textrm{sgn}_-(\tilde{\textsf{u}}(t,\textbf{x})-\lambda ) +{{\,\textrm{div}\,}}_{\textbf{x}} \big ( g'(\lambda ) \textrm{sgn}_-(\tilde{\textsf{u}}(t,\textbf{x})-\lambda )\big ) =\partial _\lambda m^-_{\tilde{\textsf{u}}}(t,\textbf{x},\lambda ), \end{aligned}$$

with initial functions $\textrm{sgn}_{+}(\tilde{u}_0(\textbf{x})-\lambda )$, $\textrm{sgn}_{-}(\tilde{\textsf{u}}_0(\textbf{x})-\lambda )$, respectively. We convolve both of the previous two equations with $\omega _{\sigma ,\epsilon ,\eta }$ and denote

$$\begin{aligned}&h^+=\textrm{sgn}_+(\tilde{u}(t,\textbf{x})-\lambda ),  &   \textsf{h}^-=\textrm{sgn}_-(\tilde{\textsf{u}}(t,\textbf{x})-\lambda ),\\&h^+_{\sigma ,\epsilon ,\eta } =\textrm{sgn}_+(\tilde{u}(t,\textbf{x})-\lambda ) \star \omega _{\sigma ,\epsilon ,\eta },  &   \textsf{h}^-_{\sigma ,\epsilon ,\eta } =\textrm{sgn}_-(\tilde{\textsf{u}}(t,\textbf{x})-\lambda ) \star \omega _{\sigma ,\epsilon ,\eta }. \end{aligned}$$

Next, we multiply the first convolved equation by $\textsf{h}^-_{\sigma ,\epsilon ,\eta }$ and the second one by $h^+_{\sigma ,\epsilon ,\eta }$, to obtain

$$\begin{aligned} \begin{aligned}&\partial _t\left( \alpha 'h^+\right) _{\sigma ,\epsilon ,\eta } \textsf{h}^-_{\sigma ,\epsilon ,\eta } +\textsf{h}^-_{\sigma ,\epsilon ,\eta } {{\,\textrm{div}\,}}_{\textbf{x}} \big ( g'(\lambda ) h^+\big )_{\sigma ,\epsilon ,\eta } \\  &\qquad =\textsf{h}^-_{\sigma ,\epsilon ,\eta } \partial _\lambda (m^+_{\tilde{u}}) _{\sigma ,\epsilon ,\eta } =\partial _\lambda \left( \textsf{h}^-_{\sigma ,\epsilon ,\eta } (m^+_{\tilde{u}}) _{\sigma ,\epsilon ,\eta }\right) \underbrace{-\partial _\lambda \textsf{h}^-_{\sigma ,\epsilon ,\eta } (m^+_{\tilde{u}}) _{\sigma ,\epsilon ,\eta }}_{\ge 0} \end{aligned} \end{aligned}$$

and

$$\begin{aligned} \begin{aligned}&\partial _t \left( \alpha '\textsf{h}^-\right) _{\sigma ,\epsilon ,\eta } h^+_{\sigma ,\epsilon ,\eta } +h^+_{\sigma ,\epsilon ,\eta } {{\,\textrm{div}\,}}_{\textbf{x}} \big ( g'(\lambda ) \textsf{h}^-\big )_{\sigma ,\epsilon ,\eta } \\  &\qquad =h^+_{\sigma ,\epsilon ,\eta } \partial _\lambda (m^-_{\tilde{\textsf{u}}}) _{\sigma ,\epsilon ,\eta } =\partial _\lambda \left( h^+_{\sigma ,\epsilon ,\eta } (m^-_{\tilde{\textsf{u}}}) _{\sigma ,\epsilon ,\eta }\right) \underbrace{-\partial _\lambda h^+_{\sigma ,\epsilon ,\eta } (m^-_{\tilde{\textsf{u}}}) _{\sigma ,\epsilon ,\eta }}_{\ge 0}, \end{aligned} \end{aligned}$$

so that

3.1 $$\begin{aligned} \begin{aligned}&\partial _t\left( \alpha 'h^+\right) _{\sigma ,\epsilon ,\eta } \textsf{h}^-_{\sigma ,\epsilon ,\eta } +\partial _t \left( \alpha '\textsf{h}^-\right) _{\sigma ,\epsilon ,\eta } h^+_{\sigma ,\epsilon ,\eta } \\  &\quad +\textsf{h}^-_{\sigma ,\epsilon ,\eta } {{\,\textrm{div}\,}}_{\textbf{x}} \big ( g'(\lambda ) h^+\big )_{\sigma ,\epsilon ,\eta } +h^+_{\sigma ,\epsilon ,\eta }{{\,\textrm{div}\,}}_{\textbf{x}} \big ( g'(\lambda ) \textsf{h}^-\big )_{\sigma ,\epsilon ,\eta } \\  &\quad \quad \ge \partial _\lambda \left( \textsf{h}^-_{\sigma ,\epsilon ,\eta } (m^+_{\tilde{u}}) _{\sigma ,\epsilon ,\eta }\right) +\partial _\lambda \left( h^+_{\sigma ,\epsilon ,\eta } (m^-_{\tilde{\textsf{u}}}) _{\sigma ,\epsilon ,\eta }\right) . \end{aligned} \end{aligned}$$

where $(\cdots )_{\sigma ,\epsilon ,\eta }$ denotes convolution of $(\cdots )$ against the kernel $\omega _{\sigma ,\epsilon ,\eta }$, see (2.8).

We multiply (3.1) by a non-negative test function $\varphi \in C^\infty _c([0,T)\times {{\mathbb {R}}}^d)$ and integrate over the domain $[0,T]\times {{\mathbb {R}}}^d\times [-M,M]$.

$$\begin{aligned}&\int _0^T \int _{{{\mathbb {R}}}^d} \int _{-M}^M \varphi (t,\textbf{x}) \left( \partial _t\left( \alpha 'h^+\right) _{\sigma ,\epsilon ,\eta } \textsf{h}^-_{\sigma ,\epsilon ,\eta } +\partial _t \left( \alpha '\textsf{h}^- \right) _{\sigma ,\epsilon ,\eta } h^+_{\sigma ,\epsilon ,\eta }\right) \, d\lambda d\textbf{x}dt \\  &\quad + \int _0^T \int _{{{\mathbb {R}}}^d} \int _{-M}^M \varphi (t,\textbf{x}) \Bigl (\textsf{h}^-_{\sigma ,\epsilon ,\eta } {{\,\textrm{div}\,}}_{\textbf{x}} \big ( g'(\lambda ) h_+ \big )_{\sigma ,\epsilon ,\eta } \\  &\qquad \qquad \qquad \qquad \qquad \qquad \quad +{h}^+_{\sigma ,\epsilon ,\eta } {{\,\textrm{div}\,}}_{\textbf{x}} \big ( g'(\lambda ) \textsf{h}^- \big )_{\sigma ,\epsilon ,\eta }\Bigr ) \, d\lambda d\textbf{x}dt \\  &\quad \quad \ge \int _0^T \int _{{{\mathbb {R}}}^d} \varphi (t,\textbf{x}) \Big (\textsf{h}^-_{\sigma ,\epsilon ,\eta }(t,\textbf{x},M) (m^+_{\tilde{u}}) _{\sigma ,\epsilon ,\eta }(t,\textbf{x},M)\\&\quad \quad \qquad \qquad \qquad \qquad -\textsf{h}^-_{\sigma ,\epsilon ,\eta }(t,\textbf{x},-M) (m^+_{\tilde{u}}) _{\sigma ,\epsilon ,\eta }(t,\textbf{x},-M) \Big ) \, d\textbf{x}dt \\  &\quad \quad \qquad -\int _0^T \int _{{{\mathbb {R}}}^d} \varphi (t,\textbf{x}) \Big ({h}^+_{\sigma ,\epsilon ,\eta }(t,\textbf{x},M) (m^-_{\tilde{\textsf{u}}}) _{\sigma ,\epsilon ,\eta }(t,\textbf{x},M)\\&\quad \quad \qquad \qquad \qquad \qquad -{h}^+_{\sigma ,\epsilon ,\eta }(t,\textbf{x},-M) (m^-_{\tilde{\textsf{u}}}) _{\sigma ,\epsilon ,\eta }(t,\textbf{x},-M)\Big ) \, d\textbf{x}dt. \end{aligned}$$

By letting $\eta \rightarrow 0$ (vanishing mollification in $\lambda$), we obtain

$$\begin{aligned} \begin{aligned}&\int _0^T \int _{{{\mathbb {R}}}^d} \int _{-M}^M \varphi (t,\textbf{x}) \left( \partial _t\left( \alpha '(\cdot ,\lambda )h^+\right) _{\sigma ,\epsilon } \textsf{h}^-_{\sigma ,\epsilon } +\partial _t\left( \alpha '(\cdot ,\lambda )\textsf{h}^- \right) _{\sigma ,\epsilon }h^+_{\sigma ,\epsilon }\right) \, d\lambda d\textbf{x}dt \\  &\qquad \quad + \int _0^T \int _{{{\mathbb {R}}}^d} \int _{-M}^M {{\,\textrm{div}\,}}_{\textbf{x}} \bigl (\varphi (t,\textbf{x})g'(\lambda ) \textsf{h}^-_{\sigma ,\epsilon } {h}^+_{\sigma ,\epsilon } \bigr ) \, d\lambda d\textbf{x}dt \qquad \big ( =0 \big ) \\  &\qquad \quad - \int _0^T \int _{{{\mathbb {R}}}^d} \int _{-M}^M \nabla _\textbf{x}\varphi \cdot g'(\lambda ) {h}^+_{\sigma ,\epsilon } \textsf{h}^-_{\sigma ,\epsilon }\, d\lambda d\textbf{x}dt \\  &\quad \ge \int _0^T \int _{{{\mathbb {R}}}^d} \varphi (t,\textbf{x}) \Big (\textsf{h}^-_{\sigma ,\epsilon }(t,\textbf{x},M) (m^+_{\tilde{u}}) _{\sigma ,\epsilon }(t,\textbf{x},M) \\  &\qquad \qquad \qquad \qquad \qquad -\textsf{h}^-_{\sigma ,\epsilon }(t,\textbf{x},-M) (m^+_{\tilde{u}}) _{\sigma ,\epsilon }(t,\textbf{x},-M) \Big ) \, d\textbf{x}dt \\  &\qquad \quad -\int _0^T \int _{{{\mathbb {R}}}^d} \varphi (t,\textbf{x}) \Big ({h}^+_{\sigma ,\epsilon }(t,\textbf{x},M) (m^-_{\tilde{\textsf{u}}}) _{\sigma ,\epsilon }(t,\textbf{x},M) \\  &\qquad \qquad \qquad \qquad \qquad -{h}^+_{\sigma ,\epsilon }(t,\textbf{x},-M) (m^-_{\tilde{\textsf{u}}}) _{\sigma ,\epsilon }(t,\textbf{x},-M)\Big ) \, d\textbf{x}dt. \end{aligned} \end{aligned}$$

As *M* approaches infinity, we can use (2.1) to derive

$$\begin{aligned} \begin{aligned}&\int _0^T \int _{{{\mathbb {R}}}^d} \int _{{{\mathbb {R}}}} \varphi (t,\textbf{x}) \left( \partial _t\left( \alpha '(\cdot ,\lambda )h^+ \right) _{\sigma ,\epsilon } \textsf{h}^-_{\sigma ,\epsilon } +\partial _t \left( \alpha '(\cdot ,\lambda )\textsf{h}^- \right) _{\sigma ,\epsilon } h^+_{\sigma ,\epsilon }\right) \, d\lambda d\textbf{x}dt \\  &\quad -\int _0^T \int _{{{\mathbb {R}}}^d} \int _{{{\mathbb {R}}}}\nabla _\textbf{x}\varphi \cdot g'(\lambda ) \, {h}^+_{\sigma ,\epsilon } \textsf{h}^-_{\sigma ,\epsilon }\, d\lambda d\textbf{x}dt \ge 0. \end{aligned} \end{aligned}$$

Recall the relation $\int _{{{\mathbb {R}}}} \alpha '(\textbf{x},\lambda ){h}^+ \textsf{h}^-\, d\lambda =-\big |\alpha (\textbf{x},\tilde{u})-\alpha (\textbf{x},\tilde{\textsf{u}})\big |_+ \in L^1([0,T]\times {{\mathbb {R}}}^d)$, see Remark 2 and condition (a) of Definition 1. Sending first $\epsilon \rightarrow 0$ (vanishing mollification in *x*) and then $\sigma \rightarrow 0$ (vanishing mollification in *t*), we obtain

3.2 $$\begin{aligned} \begin{aligned}&-\int _0^T \int _{{{\mathbb {R}}}^d} \int _{{{\mathbb {R}}}} \partial _t \varphi (t,\textbf{x}) \alpha '(\textbf{x},\lambda ) {h}^+ \textsf{h}^-\, d\lambda d\textbf{x}dt \\  &\qquad + \int _{{{\mathbb {R}}}^d} \int _{{{\mathbb {R}}}} \varphi (T,\textbf{x}) \alpha '(\textbf{x},\lambda ){h}^+(T,\textbf{x},\lambda ) \textsf{h}^-(T,\textbf{x},\lambda ) \, d\lambda d\textbf{x}\\  &\qquad -\int _{{{\mathbb {R}}}^d} \int _{{{\mathbb {R}}}} \varphi (0,\textbf{x}) \alpha '(\textbf{x},\lambda ) {h}^+(0,\textbf{x},\lambda ) \textsf{h}^-(0,\textbf{x},\lambda ) \, d\lambda d\textbf{x}\\  &\qquad -\int _0^T \int _{{{\mathbb {R}}}^d} \int _{{{\mathbb {R}}}} \nabla _\textbf{x}\varphi \cdot g'(\lambda ) \, {h}^+\textsf{h}^- \, d\lambda d\textbf{x}dt\ge 0, \end{aligned} \end{aligned}$$

where the third integral is zero if $\tilde{u}_0=\tilde{\textsf{u}}_0$.

Take $\varphi \in C_c([0,\infty )\times {{\mathbb {R}}}^d)$ and let $T\rightarrow \infty$ in (3.2). Then, by performing the integrations in $\lambda$, we obtain

$$\begin{aligned} \begin{aligned}&\int _{{{\mathbb {R}}}_+} \int _{{{\mathbb {R}}}^d}\partial _t \varphi \, \big |\alpha (\textbf{x},\tilde{u}(t,\textbf{x})) -\alpha (\textbf{x},\tilde{\textsf{u}}(t,\textbf{x})) \big |_+\, d\textbf{x}dt \\  &\qquad -\int _{{{\mathbb {R}}}^d}\varphi (0,\textbf{x})\,\big |\alpha (\textbf{x},\tilde{u}_0(\textbf{x})) -\alpha (\textbf{x},\tilde{\textsf{u}}_0(\textbf{x})) \big |_+ \, d\textbf{x}\\  &\qquad + \int _{{{\mathbb {R}}}_+} \int _{{{\mathbb {R}}}^d} \nabla \varphi \cdot \textrm{sgn}_+(\tilde{u}(t,\textbf{x})-\tilde{\textsf{u}}(t,\textbf{x})) \bigl (g(\tilde{u}(t,\textbf{x}))-g(\tilde{\textsf{u}}(t,\textbf{x}))\bigr ) \, d\textbf{x}dt\ge 0. \end{aligned} \end{aligned}$$

By the change of variables

$$\begin{aligned} \begin{aligned}&\alpha (\textbf{x},\tilde{u}(t,\textbf{x}))=u(t,\textbf{x}) \implies \tilde{u}(t,\textbf{x})=\beta (\textbf{x},u(t,\textbf{x}))\\&\alpha (\textbf{x},\tilde{\textsf{u}}(t,\textbf{x}))=\textsf{u}(t,\textbf{x}) \implies \tilde{\textsf{u}}(t,\textbf{x})=\beta (\textbf{x},\textsf{u}(t,\textbf{x})), \end{aligned} \end{aligned}$$

we deduce

$$\begin{aligned} \begin{aligned}&\int _{{{\mathbb {R}}}^d} \int _{{{\mathbb {R}}}^+} \partial _t \varphi \, \big |u(t,\textbf{x})-\textsf{u}(t,\textbf{x}) \big |_+ \, d\textbf{x}-\int _{{{\mathbb {R}}}^d} \varphi (0,\textbf{x}) \,\big | u_0(\textbf{x})-\textsf{u}_0(\textbf{x}) \big |_+ \, dt d\textbf{x}\\  &\quad + \int _{{{\mathbb {R}}}^d} \int _{{{\mathbb {R}}}^+} \nabla \varphi \cdot \textrm{sgn}_+\bigl (\beta (\textbf{x},u(t,\textbf{x})) -\beta (\textbf{x},\textsf{u}(t,\textbf{x}))\bigr )\\&\quad \qquad \qquad \qquad \qquad \qquad \times \bigl (g(\beta (\textbf{x},u(t,\textbf{x}))) -g(\beta (\textbf{x},\textsf{u}(t,\textbf{x})))\bigr ) \, dt d\textbf{x}\ge 0, \end{aligned} \end{aligned}$$

i.e., since $\beta$ is increasing in $\lambda$,

$$\begin{aligned}&\int _{{{\mathbb {R}}}^d} \int _{{{\mathbb {R}}}^+} \partial _t \varphi \, \big |u(t,\textbf{x})-\textsf{u}(t,\textbf{x}) \big |_+ \, d\textbf{x}-\int _{{{\mathbb {R}}}^d} \varphi (0,\textbf{x})\,\big | u_0(\textbf{x}) -\textsf{u}_0(\textbf{x}) \big |_+ \, dt d\textbf{x}\\  &\nonumber \quad + \int _{{{\mathbb {R}}}^d} \int _{{{\mathbb {R}}}^+} \nabla \varphi \cdot \textrm{sgn}_+\bigl (u(t,\textbf{x})-\textsf{u}(t,\textbf{x})\bigr ) \bigl ({\mathfrak {f}}(\textbf{x},u(t,\textbf{x}))-{\mathfrak {f}}(\textbf{x},\textsf{u}(t,\textbf{x}))\bigr ) \, dt d\textbf{x}\ge 0. \end{aligned}$$

Under the assumption that condition (iv) is satisfied, the remainder of the proof adheres to a conventional method, akin to that presented in [14, Theorem 3], which addresses a more intricate scenario.

Alternatively, the completion of the proof can be achieved by recognizing that condition (iv) suggests the following relationship between the functions $\alpha$ and *g*:

3.3 $$\begin{aligned} \frac{|g'(\lambda )|}{\alpha '(\textbf{x},\lambda )} \le C, \end{aligned}$$

where *C* is a constant. To see this, recall that $\mathfrak {f}(\textbf{x},\lambda ) = g(\beta (\textbf{x},\lambda ))$ and consider that $\alpha$ and $\beta$ are inverse functions, satisfying $\beta (\textbf{x},\alpha (\textbf{x},\lambda ))=\lambda$. Differentiating $\mathfrak {f}$ in $\lambda$ yields $\mathfrak {f}'(\textbf{x},\lambda ) = g'(\beta (\textbf{x},\lambda )) \beta '(\textbf{x},\lambda )$. Using the relation $\beta '(\textbf{x}, \alpha (\textbf{x}, \lambda )) \alpha '(\textbf{x}, \lambda ) = 1$, we obtain $\beta '(\textbf{x},\lambda )= \beta '(\textbf{x}, \alpha (\textbf{x}, \beta (\textbf{x},\lambda ))) = \frac{1}{\alpha '(\textbf{x}, \beta (\textbf{x},\lambda ))}$. With $\bar{\lambda }=\beta (\textbf{x}, \lambda )$,

$$ \mathfrak {f}'(\textbf{x},\lambda ) = \frac{g'(\bar{\lambda })}{\alpha '(\textbf{x},\bar{\lambda })}. $$

Taken in conjunction with condition (iv), this culminates in (3.3).

With this foundation, we return to (3.2). Employing (3.3), we deduce that

$$\begin{aligned}&\Big |\int _0^T\int _{{{\mathbb {R}}}^d} \int _{{\mathbb {R}}}\nabla _\textbf{x}\varphi \cdot g'(\lambda ) h^+\textsf{h}^- \, d\lambda d\textbf{x}dt \Big | \\  &\qquad \le C \Vert \nabla \varphi \Vert _{\infty } \int _0^T\int _{{{\mathbb {R}}}^d} \int _{{\mathbb {R}}}-\alpha '(\textbf{x},\lambda ) h^+\textsf{h}^{-} \, d\lambda d\textbf{x}dt \\  &\qquad =C \Vert \nabla \varphi \Vert _{\infty } \int _0^T\int _{{{\mathbb {R}}}^d} \bigl |\alpha (\textbf{x},\tilde{u}(t,\textbf{x})) -\alpha (\textbf{x},\tilde{\textsf{u}}(t,\textbf{x}))\bigr |_+ \, d\lambda d\textbf{x}dt, \end{aligned}$$

for some constant $C>0$. Considering the $L^1$-integrability of both $\alpha (\textbf{x},\tilde{u})$ and $\alpha (\textbf{x},\tilde{\textsf{u}})$, we can straightforwardly take the limit as $\varphi$ converges to $\textbf{1}_{[0,t]}\textbf{1}_{\mathbb {R}^d}$ in (3.2), while ensuring that $\Vert \nabla \varphi \Vert _{\infty }$ converges to 0. This yields

$$\int _{{{\mathbb {R}}}^d} \bigl |\alpha (\textbf{x},\tilde{u}(t,\textbf{x})) -\alpha (\textbf{x},\tilde{\textsf{u}}(t,\textbf{x}))\bigr |_+\, d\textbf{x}\le \int _{{{\mathbb {R}}}^d} \bigl |\alpha (\textbf{x},\tilde{u}_0(\textbf{x})) -\alpha (\textbf{x},\tilde{\textsf{u}}_0(\textbf{x}))\bigr |_+ \, d\textbf{x},$$

for a.e. $t>0$. The proof is completed by interchanging the roles of $\tilde{u}$ and $\tilde{\textsf{u}}$, and specifying that the initial functions coincide. $\square$
